# Effect of arsenic trioxide on the Tregs ratio and the levels of IFN-γ, IL-4, IL-17 and TGF-β1 in the peripheral blood of severe aplastic anemia patients

**DOI:** 10.1097/MD.0000000000020630

**Published:** 2020-06-26

**Authors:** Juanjuan Zhao, Yongping Song, Lina Liu, Shiwei Yang, Baijun Fang

**Affiliations:** Henan Key Lab of Experimental Haematology, Henan Center of Excellence in Tissue Engineering, Department of Hematology, Henan Cancer Hospital, Henan Cancer Hospital affiliated to Zhengzhou University, Zhengzhou University, Zhengzhou, China.

**Keywords:** aplastic anemia, arsenic trioxide, immune regulation, Tregs

## Abstract

Previous studies have suggested that the anticancer agent, arsenic trioxide (ATO), could attenuate T cell mediated immunity by not only inhibiting the proliferative response of T cells but by also increasing the frequency of regulatory T cells (Tregs). Furthermore, ATO represents a reasonable salvage treatment in some patients with refractory severe aplastic anemia (SAA). The current study aimed to evaluate the function of ATO on the Tregs percentage and cytokines changes in the peripheral blood mononuclear cells (PBMCs) of SAA patients.

PBMCs were collected from 20 newly diagnosed SAA patients in Henan Cancer Hospital and treated with different concentrations of ATO (0, 1, 2.5, and 5 μmol/L). Then we investigated the efficacy of ATO on Tregs ratio and the levels of interferon (IFN)-γ, interleukin (IL)-4, IL-17 and transforming growth factor (TGF)-β1 in the peripheral blood of SAA patients in vitro.

The results showed that ATO significantly increased the proportion of Tregs (*P* < .001) at 2.5 and 5 μmol/L concentrations, and the proportion of Tregs was increased with increasing ATO concentration (r = 0.524). At 1 (*P* = .03), 2.5 (*P* < .001) and 5 μmol/L (*P* < .001), ATO significantly up-regulated the expression levels of Foxp3 mRNA, which was positively and linearly correlated with the increase of Tregs cell-frequency (*r* = 0.52, 95%CI, 0.37–0.67). In addition, ATO significantly reduced the levels of IFN-γ (at 1, 2.5 and 5 μmol/L, *P* < .001), IL-4 (at 2.5 μmol/L, *P* = .009; at 5 μmol/L, *P* < .001), and IL-17 (at 2.5, *P* = .016; at 5 μmol/L, *P* < .001). ATO significantly reduced the levels of TGF-β1 at 5 μmol/L (*P* = .03), but showed no significant effects at 1 and 2.5 μmol/L (*P* > .05).

ATO could mediate the immune regulation, which might contribute to improve hematopoietic recovery in SAA patients.

## 1 Introduction

Acquired aplastic anemia (AA) is a type of myeloid hematopoietic failure, and is characterized by cytopenia in the bone marrow and peripheral blood.^[1,2]^ The current study finidngs revealed that AA is an autoimmune disease that is caused by over-activation of T-cells primarily targeting the bone marrow.^[1,3,4]^ Over-activated CD8^+^ cytotoxic T cells attack hematopoietic stem and progenitor cells, resulting in excessive apoptosis.^[5,6]^ Simultaneously, the polarization of CD4^+^ helper T (Th) cells leads to a significant increase in the levels of hematopoietic negative regulatory cytokines, such as interferon (IFN)-γ, tumor necrosis factor (TNF)-α and interleukin (IL)-2, significantly inhibiting the hematopoietic function.^[7–9]^ Th17 cells, which mediate inflammation, also play an important role in this process.^[10]^ CD4^+^CD25^+^ Tregs can inhibit autologous T cell function and induce immune tolerance.^[11,12]^ Several studies have indicated that the decrease in the number and frequency of Tregs in bone marrow and peripheral blood and the functional abnormalities play an important role in the pathogenesis and development of AA.^[13–16]^ Therefore, immunotherapy targeting regulatory T cells (Tregs) could be an effective strategy for the treatment of severe AA (SAA). Previous studies have shown that arsenicals had an immunomodulatory effect^[17]^ and low-dose arsenic exposure altered the distribution of T-cell subsets (such as CD4^+^, CD8^+^, Th1, Th2, Th17, and Tregs) and related cytokines in the peripheral blood.^[18–22]^ Recently, animal studies have demonstrated that ATO up-regulated the proportion of Tregs and inhibited T-cell immunity both in vivo and in vitro.^[23,24]^ However, there is no study till date that investigated whether ATO can increase the proportion of Tregs in the peripheral blood and inhibit T-cell immunity to promote hematopoietic recovery in SAA patients. Hence, to investigate this issue, we treated the peripheral blood mononuclear cells (PBMCs) of SAA patients with different concentrations of ATO (0, 1, 2.5 and 5 μmol/L) and detected the frequency of Tregs, levels of FxoP3 mRNA and concentrations of IFN-γ, IL-4, IL-17, and TGF-β1 to verify the results.

## 2 Materials and methods

### 2.1 Isolation of PBMCs and treatment with different concentrations of ATO in vitro

The protocol of this study followed the requirements of Declaration of Helsinki and was approved by the Ethics Committee of Henan Cancer Hospital affiliated to Zhengzhou University, China, and the institutional review board of Zhengzhou University ethics committee. Also written informed consent forms were obtained from all patients. A total of 20 newly diagnosed SAA patients, including 13 females and 7 males, with a median age of 34 (13–56) were enrolled. All the patients met the diagnostic criteria of SAA.^[25,26]^

Peripheral venous blood (50 ml) was obtained and heparinized from the patients with SAA. PBMCs were separated by a Ficoll-Paque gradient centrifugation (specific gravity 1.077 g/ml; Nycomed Pharma AS, Oslo, Norway) from 30 ml of blood sample. Then the PBMCs were washed with PBS and then resuspended in RPMI1640 medium (Gibco Life Technologies, Paisley, UK) containing 10% fetal calf serum (FCS, Gibco), 100 U/ml penicillin (Gibco), 1000 U/ ml streptomycin (Gibco), 2.5 IU/ml IL-2 (Sigma, St. Louis, MO, USA) and different concentrations ATO (0, 1, 2.5, and 5 μmol/L; Beijing SL Pharmaceutical Co. Ltd., China). This was followed by inoculation of the cells in 12-well plates at a density of 10^6^ cells/ml. PBMCs were isolated in the same way from the remaining 20 ml of blood sample for the detection of FxoP3 mRNA levels. The cells were resuspended in the medium mentioned above and then inoculated in 75 cm^2^ flasks. All the cells were incubated in 5% CO_2_/air at 37°C for 96 hours.

### 2.2 Analysis of cytotoxicity and cell apoptosis of ATO on PBMCs

Cytotoxicity of ATO on PBMCs was first evaluated using CCK-8 methods. Briefly, 196 μl of this PBMCs suspension was loaded into a 96-well round-bottomed plate at the density of 10^6^ cells/ml. Then cells were treated with 4 μl ATO (1, 2.5, and 5 μmol/L) for 96 hours and incubated with 20 μl CCK-8 reagents for another 3 hours at 37°C. The optical density (OD) was detected at a wavelength of 450 nm on Multimode Plate Readers (Tecan, Mannedorf, Switzerland). The OD values for the experimental groups are represented as the percentage change compared with control.

For the cell apoptosis analysis, cells were harvested and washed with ice-cold PBS, and resuspended in 500 μl of binding buffer. Then cells were stained with annexin V-FITC (3.5 μl) and a PI solution (2.5 μl) for 15 minutes in the dark at room temperature (KeyGen, Nanjing, China). Flow cytometry analysis was performed using a FACScan flow cytometer (BD Biosciences, USA) and CellQuest software (Becton Dickinson, USA).

### 2.3 Analysis of the proportion of CD4^+^CD25^+^CD127^low^ and CD4^+^CD25^+^FoxP3^+^ Tregs

The proportion of Tregs was analyzed by flow cytometry. PBMCs treated with different concentrations of ATO (0, 1, 2.5, and 5 μmol/L) were stained according to the manufacturers instructions. After washing twice in PBS, the cells were resuspended in PBS at a density of 10^6^/ml. The experimental groups were added with 5 μl/10^6^ cells of human CD4-FITC, CD25-APC, and CD127-PE (Franklin Lakes, NJ, USA), respectively, for the detection of CD4^+^CD25^+^CD127^low^ Tregs. For the detection of CD4^+^CD25^+^FoxP3^+^ Tregs, the other experimental groups were supplemented with 5 μl/10^6^ of CD4-FITC and CD25-APC, followed by incubation for 30 minutes in the dark after the addition of fixative and rupture agent according to the manufacturers instructions. After washing and resuspension, the cell suspensions were added with 5 μl/10^6^ cells of FoxP3-PE (Biolegend, San Diego, CA) and incubated again in the dark for 30 minutes. The control groups were added with 5 μl/10^6^ cells of FITC, APC and PE Mouse IgG1 κ Isotype control, respectively. The cells were washed with PBS twice after incubating in the dark for 30 minutes and then the supernatant was discarded. The cells were resuspended in 0.5 ml PBS for the analysis of flow cytometry. More than 10^5^ living PBMCs were collected in each specimen and the data was obtained by FACScan flow cytometer (BD Biosciences, USA). The obtained data was analyzed by using CellQuest software (Becton Dickinson, USA).

### 2.4 Analysis of the expression levels of FoxP3 mRNA

Total RNA was prepared with Trizol reagents according to manufacturers protocol (Gibco) and visualized by UV spectrophotometer. The first-strand cDNA was synthesized from 4 μg of total RNA primed with oligo-dT(18) primer using First-Strand cDNA Synthesis Kit (Gibco). The expression levels of FoxP3 mRNA were detected by RT-PCR analysis and all the specimens were normalized against the internal control β-actin. The primer sequences of FoxP3 were as follows: upstream 5’-TCAGTCCACTTCACCAAG-3’; downstream 5’-TTGAGGGAGAAGACCCCAGT-3’. The primer sequences of β-actin were as follows: upstream 5’-CTGGGACGACATGGAGAAAA-3’; downstream 5’-AAGGAAGGCTGGAAGAGTGC-3′. The relative expression levels of genes were calculated using 2^-ΔΔCt^ method.

### 2.5 Isolation and culture of macrophages in the peripheral blood from SAA patients

The peripheral venous blood (5 ml) of SAA patients was obtained, and heparin was added for anticoagulation. PBMCs were isolated by Ficoll density gradient centrifugation. The number of cells was adjusted to 3 × 10^6^/ml with IMDM medium containing 10% FCS and then cultured in a 6-well plate. After adhering for 3 hours in 5% CO_2_ incubator at 37°C, the unadhered cells were washed away with pre-warmed medium. 2 ml of IMDM medium containing 10% FCS and rhGM-CSF (final concentration 1000 μ/Ml; Peprotech, USA) was added, and then the solution was changed after every 2.5 days. After 7 days of culture, the adherent cells were identified as macrophages. The positive rate of CD14 cells was 85.32% (±7.68), as indicated by flow cytometry. The adherent cells were collected and then seeded in a 6-well plate. After treatment with different concentrations of ATO (0, 1, 2.5, and 5 μmol/L) for 96 hours, the supernatant was collected, and the concentration of TGFβ1 in the supernatant was determined by enzyme linked immunosorbent assay (ELISA).

### 2.6 Analysis of the concentrations of IFN-γ, IL- 4, IL17 and TGF-β1

Cytokines were analyzed by ELISA kit (PeproTech EC Ltd, London, UK) ccording to the manufacturer's instructions. The supernatant of the culture medium with different concentrations ATO (0, 1, 2.5, and 5 μmol/L) was collected and temporarily stored at −80^o^C in a refrigerator for testing. Implementation procedures were carried out according to the manufacturers instructions.

### 2.7 Statistical analysis

SPSS. 17.0 software (SPSS, Statistical Product and Service Solutions, Chicago, USA) was used for statistical analysis and multiple group comparisons were performed by one-way ANOVA, Tukeys post-hoc analysis to compare the results between the groups treated with different concentrations of ATO (0, 1, 2.5, and 5 μmol/L). The correlations between the proportion of Tregs and concentrations of ATO and levels of FoxP3 mRNA were detected by Pearson correlation analysis, respectively. All the results were presented as means ± standard deviation (X ± S), and *P* value of <.05 was considered to be statistically significant.

## 3 Results

### 3.1 ATO treatment reduced PBMCs viability and increased the cell apoptotic rate

**Figure 1:**
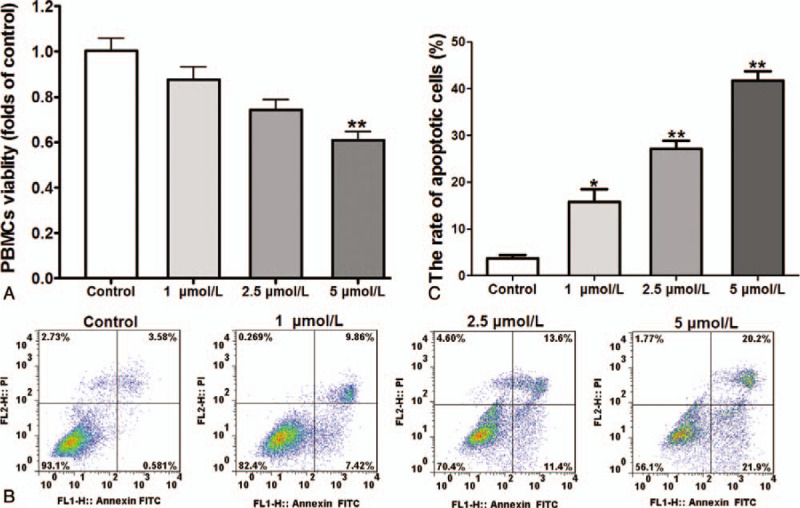
Analysis of cytotoxicity and cell apoptosis of ATO on PBMCs. (A) Cell viability was determined by the CCK-8 assay and is expressed as the mean ± SD of 3 separate experiments.^∗∗^*P* < .01 versus control. (B) Flow cytometry analysis of the viability of PBMCs cells in different group. The results from 1 of the 3 independent experiments are shown. (C) Quantification of the percentage of living cells in each group. Error bars represent mean ± SEM. ^∗^*P* < .05, ^∗∗^*P* < .01 vs control group.

As shown in Fig. 1A, with the ATO concentration increased, the PBMCs viabilities were significantly decreased. 5 μmol/L ATO treatment caused nearly 40% cell viability reduce compared with the control group. Furthermore, ATO induced PBMCs death was analyzed by Flow cytometry. Results showed that, ATO significantly increased the apoptotic rates (including early apoptosis and late apoptosis) in PBMCs, whereas the living cells were significantly reduced with the increasing ATO concentrations (Fig. 1B and C).

### 3.2 ATO upregulated the proportion of CD4^+^CD25^+^CD127^low^ Tregs and CD4^+^CD25^+^FoxP3^+^ Tregs in SAA patients

**Figure 2:**
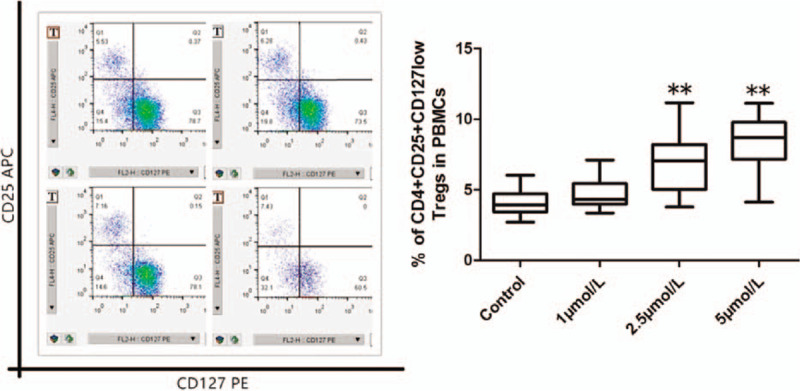
The proportion of CD4^+^CD25^+^CD127^low^ Tregs in PBMCs from SAA patients treated with different concentrations of ATO (0, 1, 2.5, and 5 μmol/L) for 96 hours by flow cytometry. ^∗^*P* < .05 and ^∗∗^*P* < .001 compared to the control group.

**Figure 3:**
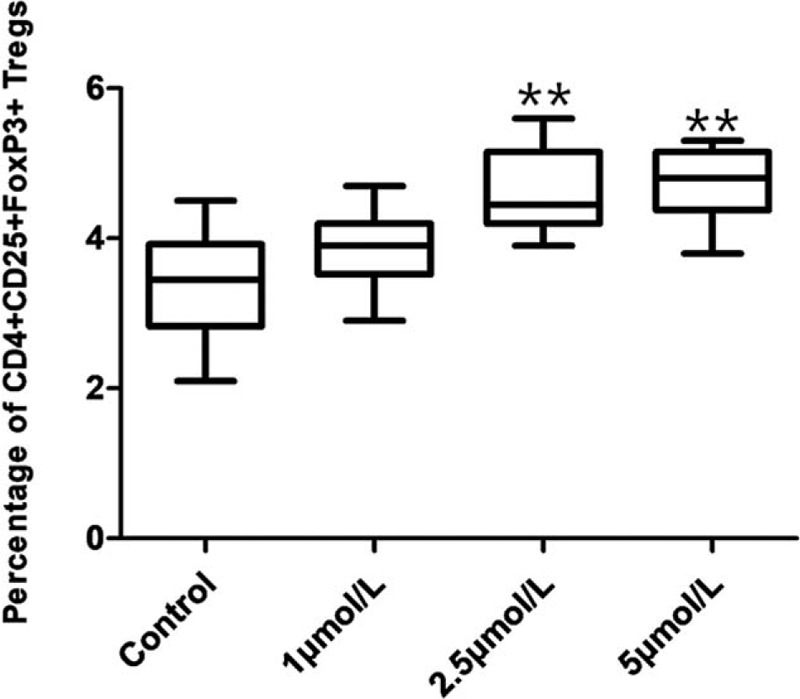
The proportion of CD4^+^CD25^+^ FoxP3^+^ Tregs in PBMCs from SAA patients treated with different concentrations of ATO (0, 1, 2.5, and 5 μmol/L) for 96 hours by flow cytometry. ^∗^*P* < .05 and ^∗∗^*P* < .001 compared to the control group. ^∗^*P* < .05 and ^∗∗^*P* < .001, compared with control group.

To investigate the effect of ATO on the proportion of CD4^+^CD25^+^CD127^low^Tregs and CD4^+^CD25^+^FoxP3^+^Tregs in vitro, we treated PBMCs from patients with SAA at different concentrations of ATO (0, 1, 2.5, and 5 μmol/L) for 96 hours and analyzed using flow cytometry. CD4^+^ cells of the lymphocyte fraction were gated, and the percentages of CD25^+^CD127^low^ or CD25^+^FoxP3^+^ cells were figured out. The results showed that ATO significantly increased (*P* < .001) the percentage of CD25^+^CD127^low^ Tregs in CD4^+^ cells at 2.5 and 5 μmol/L concentrations, and the degree of CD4^+^CD25^+^CD127^low^ Tregs was increased with the addition of ATO concentration (*r* = 0.524). At 1 μmol/L, ATO increased the percentage of CD4^+^CD25^+^CD127^low^ Tregs in CD4^+^ cells; however, the increase was not statistically significant (*P* = .052; Fig. 2). In addition, the results showed that ATO significantly increased (*P* < .001) the percentage of CD4^+^CD25^+^FoxP3^+^Tregs in CD4^+^ cells at 2.5 and 5 μmol/L concentrations. At 1 μmol/L, ATO increased the percentage of Tregs in CD4^+^ cells, but the increase was not statistically significant (*P* = .137; Fig. 3). The changing trend in the percentage of Tregs detected by CD4^+^CD25^+^FoxP3^+^ was consistent with CD4^+^CD25^+^CD127^low^, but the level of Tregs detected was lower, contributing to complicated operations such as membrane rupture during the labeling process.

### 3.3 ATO increased the expression levels of FxoP3 mRNA in cultured PBMCs from SAA patients

**Figure 4:**
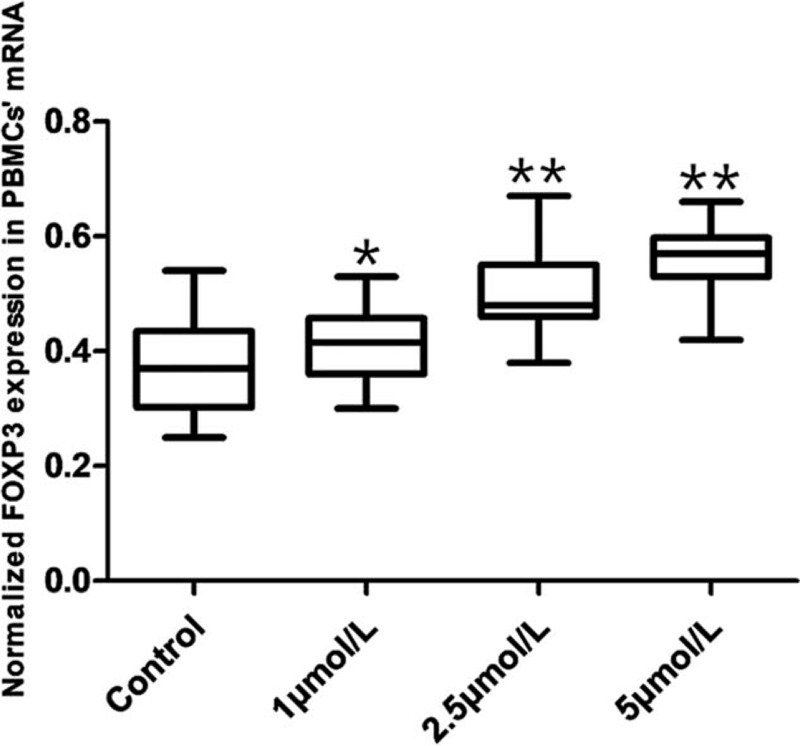
The expression levels of Foxp3 mRNA in PBMCs from SAA patients treated with different concentrations of ATO (0, 1, 2.5, and 5 μmol/L) for 96 hours by RT-PCR. ^∗^*P* < .05 and ^∗∗^*P* < .001 compared to the control group.

After treating PBMCs of SAA patients with different concentrations of ATO (0, 1, 2.5, and 5 μmol/L) for 96 hours, 10^7^ viable cells were counted by trypan blue for the detection of FoxP3 mRNA levels by Q-PCR. The results showed that ATO significantly increased the expression levels of FxoP3 mRNA at 1 (*P* = .03), 2.5 (*P* < .001) and 5 μmol/L (*P* < .001) (Fig. 4).

### 3.4 Correlation of the expression levels of FoxP3 mRNA with the percentage of CD4^+^CD25^+^CD127^low^ Tregs

Pearson correlation was performed to analyze the correlation between FxoP3 mRNA expression levels and CD4^+^CD25^+^CD127^low^ Tregs percentage. Statistical data have shown that Pearson product-moment correlation coefficient *r* = 0.52 (*t* = 5.42, *P* < .001, 95%CI = 0.37–0.67). The results demonstrated that the expression levels of FoxP3 mRNA were positively correlated with the percentage of CD4^+^CD25^+^CD127^low^ Tregs.

### 3.5 Effects of ATO on cytokines in cultured PBMCs from SAA patients

**Figure 5:**
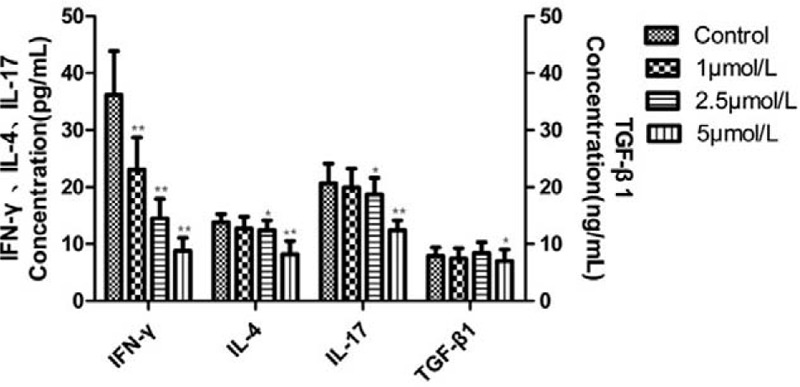
Effects of different concentrations of ATO (0, 1, 2.5 and 5 μmol/L) on the levels of IFN-γ, IL-4, IL-17 and TGF-β1 in PBMCs culture supernatant. ^∗^*P* < .05 and ^∗∗^*P* < .001 compared to the control group.

ELISA was performed to detect the concentrations of cytokines in the supernatant of cultured PBMCs treated with different concentrations of ATO (0, 1, 2.5, and 5 μmol/L) for 96 hours. The results showed that ATO significantly reduced (*P* < .001) the concentrations of IFN-γ at 1 (23.08 ± 7.67), 2.5 (14.50 ± 3.42) and 5 μmol/L (8.76 ± 2.29). Similarly, ATO significantly inhibited the expression levels of IL-4 at 2.5 (12.43 ± 1.70; *P* = .009) and 5 μmol/L (8.20 ± 2.32; *P* < .001), and the concentrations of IL-17 were significantly reduced at 2.5 μmol/L (18.69 ± 2.97; *P* = .016) and 5 μmol/L (12.43 ± 1.70; *P* < .001). The levels of TGF-β1 were not significantly affected at 1 μmol/L (7.45 ± 1.80; *P* = .45) and 2.5 μmol/L (8.40 ± 1.90; *P* = .35), while the concentrations of TGF-β1 were significantly decreased at 5 μmol/L (6.72 ± 1.90; *P* = .03), (Fig. 5).

### 3.6 Effects of ATO on TGFβ1 concentration secreted by macrophages from SAA patients

**Figure 6:**
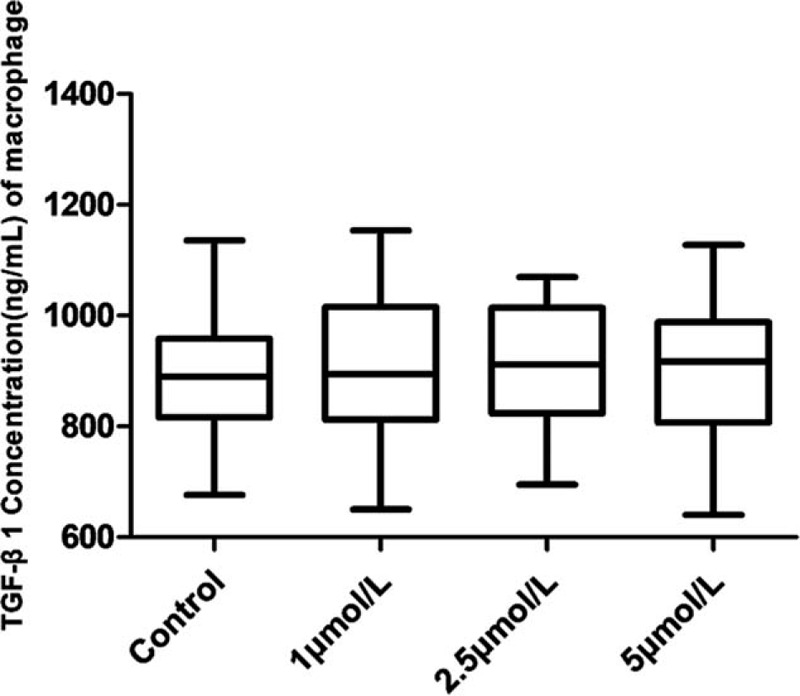
Effects of different concentrations of ATO (0, 1, 2.5 and 5 μmol/L) on the levels of TGF-β1 secreted by macrophages from SAA patients. ^∗^*P* < .05 and ^∗∗^*P* < .001 compared to the control group.

ELISA was performed to detect the concentrations of TGF-β1 secreted by macrophages of SAA patients treated with different concentrations of ATO (0, 1, 2.5, and 5 μmol/L) for 96 h. The results showed that ATO had no significant effect on TGFβ1 secreted by macrophages compared with the control group (Fig. 6).

## 4 Discussion

At present, several studies have demonstrated the immunoregulatory effects of ATO, such as upregulation of the number and percentage of Tregs both in vitro and in vivo.^[27]^ The decrease in the number and percentage of Tregs play an important role in the pathogenesis and development of AA.^[13]^ However, there is no evidence available on till date regarding the immunoregulatory effects of ATO on Tregs in patients with AA.

Recently, the results of our small sample study found that the overall complete response rate and overall response rate at 17 weeks reached up to 60% and 100% in 5 patients with refractory SAA who received ATO alone at a dose of 0.15 mg/kg intravenously daily for 5 consecutive days.^[28]^ In addition, we administered ATO plus cyclosporine in patients with SAA. The overall complete response rate and overall response rate at 17 weeks after the initiation of treatment were 80% (8/10) and 100% (10/10), respectively.^[29]^ So treatment with ATO may be a novel therapeutic approach for SAA. Our further study found that ATO could significantly improve the microenvironment of bone marrow hematopoiesis in SAA patients by partially restoring the differentiation imbalance of bone marrow mesenchymal stem cells,^[30]^ providing a possible mechanism for explaining the effectiveness of ATO in the treatment of SAA. However, is there any other mechanism for ATO to effectively treat SAA? For example, what about immune regulation?

In fact, toxicological studies have shown that long-term exposure of ATO can suppress T-cell immunity. Gera et al^[24]^ have demonstrated that chronic long-term exposure of ATO inhibited T-cell function and reduced the secretion of relevant cytokines that is associated with a significant increase of CD4+CD25+ Tregs in animal model. Studies have shown that ATO exposure during early development of immune system can induce thymus atrophy and inhibit T-cell immunity in children, thereby resulting in an increased risk of infection.^[31,32]^

To the best of our knowledge, this is the first study to investigate the immunoregulatory effect of ATO in patients with newly-diagnosed SAA. Our results have demonstrated that ATO (2.5 and 5 μmol/L) could increase the percentage of Tregs in the peripheral blood of SAA patients, and the results were confirmed by flow cytometry detection of increased FoxP3 protein levels and RT-PCR detection of increased FoxP3 mRNA expression levels. ELISA tests results showed that Th1, Th2, and Th17 related cytokines were reduced, indicating that ATO reduced the other T-cell subsets. TGF-β1 has a relatively positive correlation with the levels of Tregs. At the same time, we ruled out the effects of TGF-β1 secreted by macrophages. ATO showed no significant effects on TGF-β1 at low concentrations (1 and 2.5 μmol/L), while the levels of TGF-β1 were significantly reduced at high concentrations (5 μmol/L). This was inconsistent with the previous study findings, showing that ATO could increase the levels of TGF-β1.^[23]^ Currently, various studies have speculated that ATO could also induce the apoptosis of Tregs; however, Tregs are highly resistant to ATO when compared with other T-cell subsets, resulting in a relatively higher percentage of Tregs.^[23]^ Furthermore, according to the previous animal experiments, small doses of long-term (30 days) ATO exposure increased the number of Tregs in the spleen of mice.^[24]^ In our study, the in vitro experiments were performed for a shorter period of time (96 hours), so the effects of ATO might be not completely revealed. Therefore, we can not rule out the possibility that ATO increases the absolute number of Tregs. Patients with AA not only have reduced number and percentage of Tregs, but also have the functional abnormalities of Tregs.^[14]^ Our results have shown that ATO reduced the concentrations of hematopoietic negative regulatory factors, such as IFN-γ and IL-17, inhibiting the function of related T cells. Therefore, ATO could compensate for the immunosuppressive function of impaired Tregs to a certain extent and inhibit T cell immunity together with increased percentage of Tregs, thereby exhibiting immunoregulatory effects in SAA patients. However, to confirm this conjecture, we need to expand the sample size of clinical studies and further conduct studies related to molecular mechanisms in vitro and in vivo.

Another limitation of our study is that, our current study more focused on function of ATO on the immunoregulatory effects in the PBMCs from SAA patients, the relationship of Tregs ratio and cytokine levels, as well as the mechanism for ATO on the Tregs percentage changes were still unclear, which need us to pay more attentions in our future work.

In summary, the results of this study and our previous research suggest that the possible mechanisms for ATO to effectively treat SAA may be at least the following 2 aspects. First, ATO can significantly improve the microenvironment of bone marrow hematopoiesis in SAA patients.^[28–30]^ Second, ATO can mediate the immune regulation by upregulating the percentage of Tregs and reducing the levels of IFN-γ, IL-4, IL-17, and TGF-β1 in the peripheral blood of SAA patients.

## Acknowledgments

The authors would like to thank all the patients for their cooperation and consent.

## Author contributions

**Data curation:** Yongping Song.

**Formal analysis:** Yongping Song.

**Funding acquisition:** Baijun Fang.

**Methodology:** Juanjuan Zhao, Lina Liu, Shiwei Yang.

**Project administration:** Baijun Fang.

**Resources:** Lina Liu.

**Software:** Yongping Song, Shiwei Yang.

**Writing – original draft:** Juanjuan Zhao, Baijun Fang.

**Writing – review & editing:** Baijun Fang.
